# A Comparative Study of Raw and Metal Oxide Impregnated Carbon Nanotubes for the Adsorption of Hexavalent Chromium from Aqueous Solution

**DOI:** 10.1155/2017/1624243

**Published:** 2017-04-10

**Authors:** Muhammad I. Qureshi, Faheemuddin Patel, Nadhir Al-Baghli, Basim Abussaud, Bassam S. Tawabini, Tahar Laoui

**Affiliations:** ^1^Department of Chemical Engineering, KFUPM, Dhahran 31261, Saudi Arabia; ^2^Department of Mechanical Engineering, KFUPM, Dhahran 31261, Saudi Arabia; ^3^Department of Geosciences, KFUPM, Dhahran 31261, Saudi Arabia

## Abstract

The present study reports the use of raw, iron oxide, and aluminum oxide impregnated carbon nanotubes (CNTs) for the adsorption of hexavalent chromium (Cr(VI)) ions from aqueous solution. The raw CNTs were impregnated with 1% and 10% loadings (weight %) of iron oxide and aluminum oxide nanoparticles using wet impregnation technique. The synthesized materials were characterized using scanning electron microscopy (SEM) and thermogravimetric analysis (TGA). Batch adsorption experiments were performed to assess the removal efficiency of Cr(VI) ions from water and the effects of pH, contact time, adsorbent dosage, and initial concentration of the Cr(VI) ions were investigated. Results of the study revealed that impregnated CNTs achieved significant increase in the removal efficiency of Cr(VI) ions compared to raw CNTs. In fact, both CNTs impregnated with 10% loading of iron and aluminum oxides were able to remove up to 100% of Cr(VI) ions from aqueous solution. Isotherm studies were carried out using Langmuir and Freundlich isotherm models. Adsorption kinetics of Cr(VI) ions from water was found to be well described by the pseudo-second-order model. The results suggest that metallic oxide impregnated CNTs have very good potential application in the removal of Cr(VI) ions from water resulting in better environmental protection.

## 1. Introduction

Chromium is mainly found in natural deposits as ores and other compounds such as chrome ochre (Cr_2_O_3_), crocoite (PbCrO_4_), and ferric chromite (FeCr_2_O_4_). It is the sixth most abundant transition metal [1, 2]. Chromium is discharged into water bodies from a number of industrial sources such as electroplating and metal cleaning, leather tanning, mining of chrome ore, production of steel and alloys, dyes and pigments, glass industry, wood preservation, and textile industry [2–5].

Chromium is found in different oxidation states such as 2+, 3+, and 6+. In water, it can exist in the form of chromate ion (CrO_4_^2−^), chromic acid (H_2_CrO_4_), hydrogen chromate ion (HCrO_4_^−^), and dichromate ion (Cr_2_O_7_^2−^) [6–8]. However, the hexavalent Cr(VI) and trivalent Cr(III) are the two most stable forms present in water in neutral pH range.

The typical concentration of chromium in industrial water ranges from 5.2 to 208,000 mg/L [9, 10]. The maximum allowable limits of chromium in drinking water are 0.05 and 0.1 mg/L, as suggested by the World Health Organization (WHO) and US Environmental Protection Agency (EPA), respectively [11–15].

Due to its carcinogenic and mutagenic nature, Cr(VI) is considered as almost 300 times more toxic than Cr(III) [16]. The toxic effects of Cr(VI) include liver and kidney damage, nausea, dermatitis, diarrhea, vomiting, internal hemorrhage, and repository problems (asthma). Eye and skin contact may cause permanent damage to eye, severe burn, irritation, ulceration, and nasal septum [17, 18].

A number of remediation techniques have been reported to get rid of the Cr(VI) from water including solvent extraction [19], floatation [20], coagulation [21], ion exchange [22–25], membrane technologies [26, 27], adsorption [6, 7, 28] and cyanide treatment [29], and reduction followed by chemical precipitation [30]. However, adsorption is the most versatile, cost effective, and widely used method for removal of different contaminants from water including heavy metals. In the literature, different adsorbents have been reported for the removal of Cr(VI) from water including anaerobic sludge [31], lignocellulosic solid wastes [32], carbon slurry [33], waste slurry [34], agricultural wastes [35], cow dung carbon [36], corncob [37], almond shell carbon [38], zeolite [39], hazelnut shell carbon [40, 41], rice Polish [42], sphagnum moss peat [43], apple residue [44], moss [45], rice husk carbon [46], fly ash [6, 47], pine needles, charcoal, wool, olive stone/cake, cactus [48], used tyre carbon [49], coconut tree sawdust carbon [50], sawdust [51], dust coal, coconut shell and wood activated carbons [52], clay [53], palm pressed fibers and coconut husk [54], activated groundnut husk carbon [55], polyaniline coated on sawdust [56], coniferous leaves [57], leaf mould [58], wheat bran [59], sugar beet pulp [60], seaweeds [61], tannin gel particles [62], seaweed biosorbent [63], chitosan-1,2-cyclohexylenedinitrilotetraacetic acid–graphene oxide (Cs/CDTA/GO) nanocomposite [64], paper mill sludge [65], hydrous concrete particles [66], waste tea [67], activated alumina, rice husk ash, neem bark, saw dust, fuller's earth [6], eucalyptus bark, activated charcoal, and charred rice husk [68], treated waste newspaper [69], and graphene oxide (GO) [70].

Recently, carbon nanotubes (CNTs) have emerged as a novel adsorbent for the removal of various contaminants from water. CNTs offer the advantages of high porous and hallow structure, light mass density, large surface, and strong interaction with the pollutant molecules [28]. Studies have confirmed that surface modification of CNTs significantly enhanced their adsorption capability for the removal of various contaminants from water [71–76].

In the present study, raw CNTs and CNTs impregnated with iron oxide and aluminum oxide nanoparticles were used for the adsorption of Cr(VI) from water. The synthesized materials were characterized using scanning electron microscopy (SEM) and thermogravimetric analysis (TGA). Batch adsorption experiments were performed and the effect of pH, contact time, adsorbent dosage, and initial concentration of the adsorbate on the removal efficiency of Cr(VI) from water was investigated. Isotherm studies were carried out using Langmuir and Freundlich isotherm models.

## 2. Experimental

### 2.1. Materials Preparation

Raw CNTs were acquired from Chengdu Organic Chemicals Co. Ltd. (China), with the following characteristics: 95% purity, outside diameter of 10–20 nm, and length ranging from 1 to 10 *μ*m. These raw CNTs were impregnated with 1% and 10% loadings (weight %) of iron oxide and aluminum oxide nanoparticles using wet impregnation technique. Specific amount of CNTs was added in ethanol and sonicated to achieve homogenous dispersion of CNTs. Specific amount of metallic salt dissolved separately in ethanol and was sonicated, and then the resultant solution was added dropwise to the CNTs dispersed in ethanol. This dispersion was sonicated for proper mixing with CNTs and subsequently heated at 80–90°C in an oven overnight to evaporate the ethanol. On complete drying, the CNTs were calcined in a furnace at 350°C for 4 hours. This process resulted in the attachment of metal oxide nanoparticles onto the surface of CNTs.

### 2.2. Characterization of the Adsorbents

Raw and impregnated CNTs were characterized using various techniques. In order to perform morphological and elemental analysis, samples were coated with about 5 nm thick layer of platinum using Quorum sputter coater (Model: Q150R S). Scanning electron microscope (Model: TESCAN MIRA 3 FEG-SEM) was used to analyze the morphology and structure of raw and metal oxide impregnated CNTs. Thermogravimetric analysis (TGA) of raw and impregnated CNTs was performed using TA Instrument (Model: SDTQ600), in order to evaluate the purity and thermal degradation of materials. Samples were heated to 900°C in air, at heating rate of 10°C/min and air flow rate of 100 mL/min.

### 2.3. Batch-Mode Adsorption Experiment

Batch experiments were performed to study the effect of various parameters on the adsorption of Cr(VI) ions by raw and metal oxide impregnated CNTs at room temperature.

The effect of pH, contact time, agitation speed, and adsorbent dosage was investigated on the removal of Cr(VI) ions from aqueous solution. Concentration of Cr(VI) ions was measured using inductively coupled plasma mass spectrometer (Thermo-Fisher, X-Series 2 Q-ICP-MS).

Percentage removal and adsorption capacity were calculated using (1) and (2), respectively:

(1) Removal  efficiency %=Co−CtCo∗100

(2) Adsorption  capacity q=Co−CtVm,

where “*C*_*o*_” is the initial concentration (ppm) at start of the experiment (*t* = 0), while “*C*_*t*_” is the concentration at time “*t*”. “*V*” is the volume (L) of the solution and “*m*” represents the amount (g) of the adsorbent dosage. For the batch adsorption experiments, the stock solution was prepared using the same methodology reported previously [73].

## 3. Results and Discussion

### 3.1. Characterization of Raw and Metal Oxide Impregnated CNTs

Surface morphologies of the raw and metal oxide impregnated CNTs were observed using SEM.

**Figure 1:**
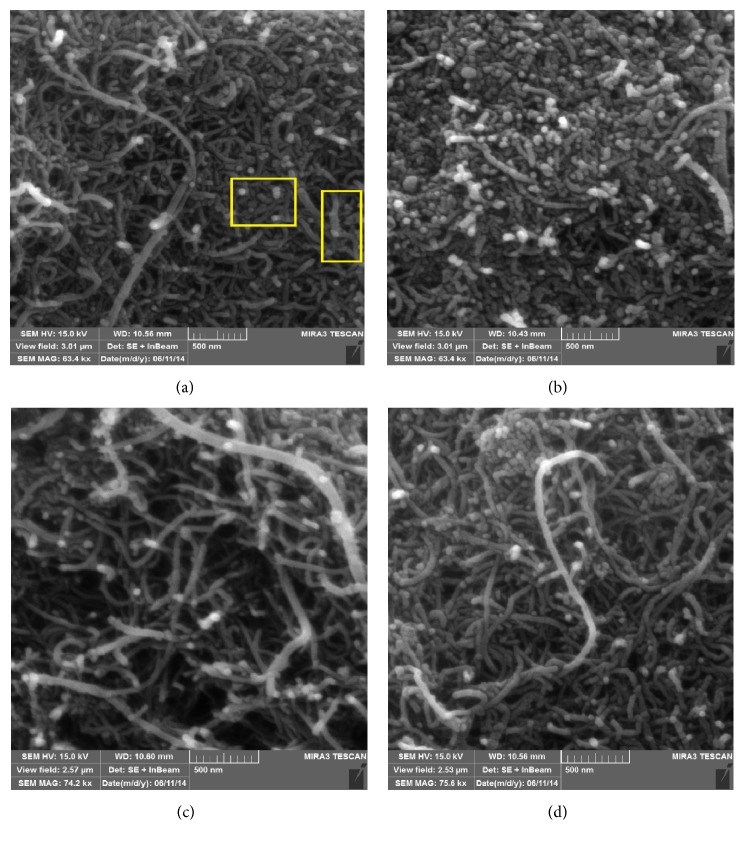
SEM images of CNTs with (a) 1% iron oxide (boxes indicate the iron oxide nanoparticles impregnated on CNTs), (b) 10% iron oxide, (c) 1% aluminum oxide, and (d) 10% aluminum oxide.

Figure 1 shows the SEM images for the metal oxides impregnated CNTs. Tubular geometry of the CNTs was observed and no damage was noticed in CNTs structures after impregnation. Metal oxide nanoparticles (highlighted in the box) were clearly observed on the surface of CNTs as displayed in Figures 1(a)–1(d). CNTs were properly dispersed for the low loading of 1% metal oxide (Figures 1(a) and 1(c)); however, at higher loading (10%) a little agglomeration of metal oxide particles could be seen in Figures 1(b) and 1(d). In general, the dispersion of CNTs was improved after impregnation with metal oxide nanoparticles. Metal oxide nanoparticles might help reduce the strong Van der Waals forces between CNTs leading to their improved dispersion.

**Figure 2:**
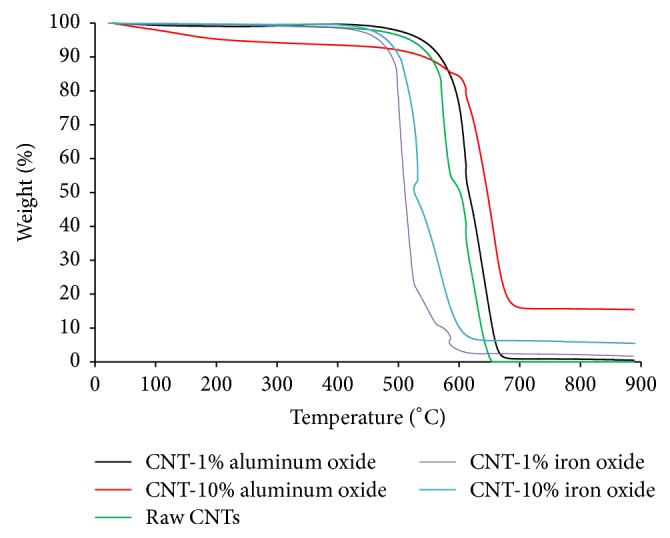
TGA curves for raw and metal oxide impregnated CNTs.

TGA curves for raw and metal oxide impregnated CNTs are presented in Figure 2. CNTs were heated to 900°C at a rate of 10°C/min under air. All the TGA curves have two main weight loss regions. Initial small weight loss was attributed to the evaporation of physically bound water and some other lighter impurities. The second, steep, and rapid weight loss region represents the combustion of CNTs. Raw CNTs showed more stability and started degrading around 550°C while degradation of 1% and 10% metal oxide impregnated CNTs started around 450°C and 500°C, respectively. This may be due to the fact that the impregnation of metal oxide nanoparticles on CNTs serves as an impurity hence leading to steep weight loss at lower temperature [77]. The weight of the residue left at the end of the analysis is the indication of metallic oxide nanoparticles. It can be observed that the amount of residue left was higher for the CNTs with 10% metal oxide loading as compared to raw CNTs and CNTs with 1% metal oxide loadings.

### 3.2. Effect of pH

**Figure 3:**
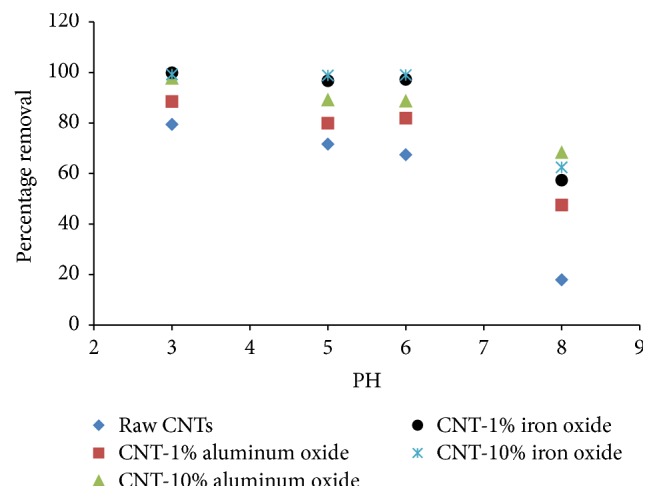
Effect of pH on the percentage removal of Cr(VI) (initial concentration = 1 mg/L, agitation speed = 200 rpm, adsorbent dosage = 200 mg, and time = 2 hours).

The removal of Cr(VI) ions by raw and metal oxide impregnated CNTs, as a function of pH, is presented in Figure 3. Solution pH was varied from 3 to 8, while the other variables including adsorbent dosage, contact time, agitation speed, and Cr(VI) initial concentration were kept constant at 200 mg, 2 hours, 50 mg, 200 rpm, and 1 mg/L, respectively.

A maximum removal of Cr(VI) was achieved at pH 3, while the removal was observed to decrease with increase in pH, for all the adsorbents. This phenomenon can be explained on the basis of surface charge of the adsorbents and ionic chemistry of the solution.

Chromium ions may exist in the form of chromate (CrO_4_^2−^), dichromate (Cr_2_O_7_^2−^), and hydrogen chromate (HCrO_4_^−^), depending upon the solution pH and chromate concentration.

The equilibrium between the chromate (CrO_4_^2−^) and dichromate ions (Cr_2_O_7_^2−^) in aqueous solution is represented by (3) [15, 73].

(3) $$\begin{array}{c} 2{CrO_{4}}^{2-}+2H^{+}⟷{{Cr}_{2}O_{7}}^{2-}+H_{2}O \end{array}$$

Chromate (CrO_4_^2−^) ions are the dominant species at high pH values, while, at low pH, mainly dichromate ions (Cr_2_O_7_^2−^) exist in the solution [78, 79].

At low pH, the high removal of Cr(VI) ions is attributed to the electrostatic interaction between the Cr_2_O_7_^2−^ anions and positively charged CNTs surface. However, at high pH, surfaces of the CNTs carry more negative charges and repulsion between the CrO_4_^2−^ ions and the CNTs surfaces resulted in lower removal of Cr(VI) ions. Furthermore, the low removal might also be due to competition between the OH^−^ and chromate (CrO_4_^2−^) ions over the limited adsorption sites as well as due to precipitation of Cr(OH)_3_ that might occur at high pH (here at pH = 8) [73].

Surface impregnation of CNTs with metal oxide was observed to enhance the removal efficiency. The maximum removal of 87.8% was obtained for CNT with 10% aluminum oxide loading at pH 3. Raw CNTs were still able to remove almost 74% Cr(VI) ions at same pH and under similar experimental conditions. Although the maximum removal was obtained at pH 3, however, to evaluate the potential of the adsorbents in real water treatment applications, a pH value of 6 was selected for the remaining experiments.

Because the solution pH has a significant effect on the removal of Cr(VI) ions, we may deduce that the main mechanism is electrostatic interaction. The net surface charge of the adsorbent changes with pH and affects the removal of Cr(VI). In addition to electrostatic interaction, some physical adsorption of Cr(VI) ions is expected on the surfaces of the CNTs due to Van der Walls interactions. Studies also suggest that strong surface complexation and ion exchange are the main mechanisms involved during the adsorption of Cr(VI) ions on CNTs surface [80].

### 3.3. Effect of Contact Time

**Figure 4:**
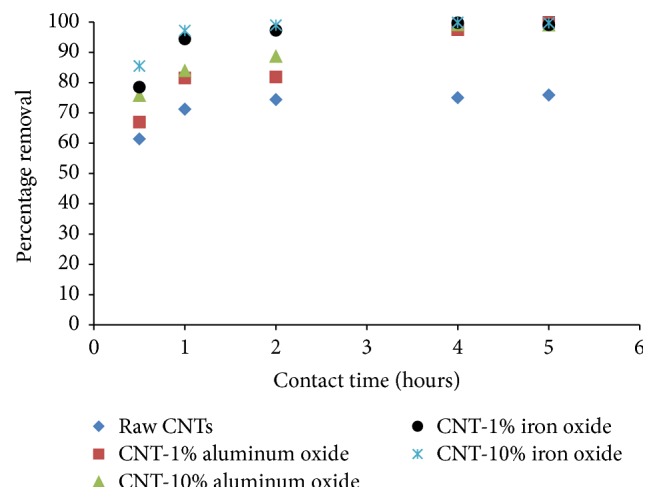
Effect of contact time on percentage removal of Cr(VI). (Initial concentration = 1 mg/L, agitation speed = 200 rpm, adsorbent dosage = 200 mg, pH = 6).

The experimental results presenting the effect of time on the removal of Cr(VI) ions by raw and metal oxide impregnated CNTs are shown in Figure 4. Contact time was varied from 0.5 to 5 hours while the solution pH, Cr(VI) initial concentration, adsorbent dosage, and agitation speed were kept constant at 6, 1 mg/L, 200 mg, and 200 rpm, respectively.

It is obvious that Cr(VI) ions removal has improved significantly as the contact time increased from 0.5 to 4 hours. No significant increase in removal was observed after 4 hours of contact time indicating the reach of equilibrium. It was observed that CNTs impregnated with metal oxide were able to remove more than 97% of Cr(VI) ions after 2 hours of contact time (for CNTs impregnated with iron oxide) and almost 100% after 4 hours of contact time (for CNTs impregnated with both iron and aluminum oxides).

### 3.4. Effect of Adsorbent Dosage

**Figure 5:**
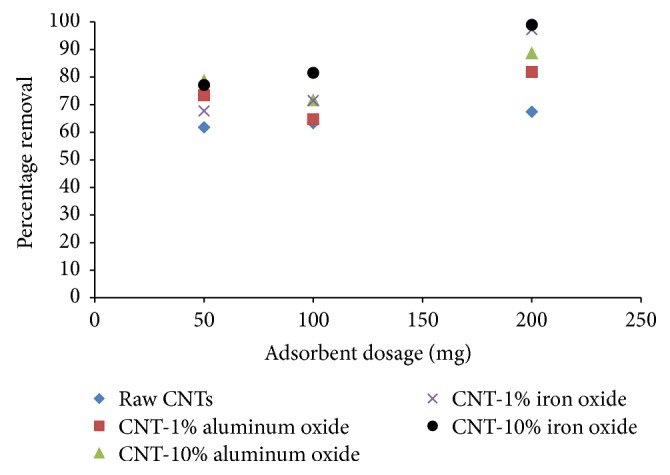
Effect of adsorbent dosage on percentage removal of Cr(VI) (initial concentration = 1 mg/L, agitation speed = 200 rpm, contact time = 2 hours, and pH = 6).

The effect of adsorbent dosage on the removal of Cr(VI) ions is depicted in Figure 5. The adsorbent dosage was varied from 50 to 200 mg, while solution pH, contact time, initial concentration of Cr(VI), and agitation speed were kept constant at 6, 2 hours, 1 mg/L, and 200 rpm, respectively.

A direct relationship was observed between the adsorbent dosage and the removal of Cr(VI) ions for all adsorbents. The removal was observed to increase with increase in the adsorbent dosage and the maximum removal was recorded at 200 mg dosage. With increase in the adsorbent dosage, the number of active sites increases; hence more Cr(VI) ions can be adsorbed onto the adsorbent surface. At 200 mg dosage, CNTs with 10% loading of iron oxide yielded a maximum removal of 99% of Cr(VI) ions, as compared to raw CNTs yielding about 67% removal under similar experimental conditions. These results confirmed that metal oxide loading has a significant effect on the removal efficiency of the raw CNTs.

### 3.5. Effect of Agitation Speed

**Figure 6:**
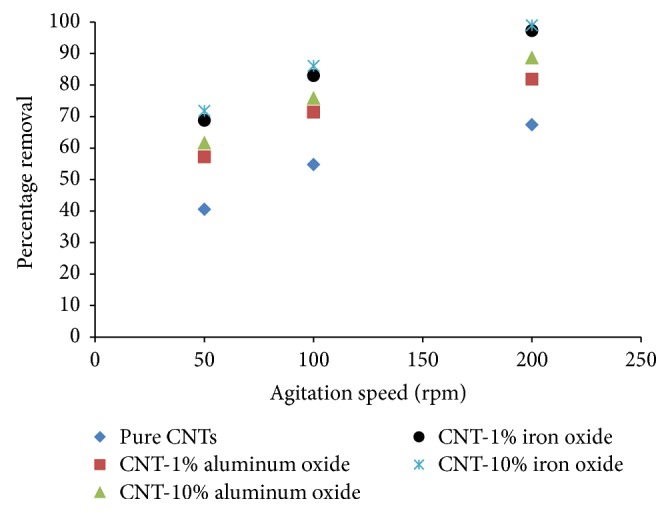
Effect of agitation speed on percentage removal of Cr(VI) ions (initial concentration = 1 mg/L, adsorbent dosage = 200 mg, contact time = 2 hours, and pH = 6).

Agitation speed is an important parameter that effects and enhances the dispersion of the adsorbent in the solution and reduces the agglomeration. For the two loadings of metal oxides (1% and 10%) used in the present study, the CNTs were found to properly disperse in the solution and no significant agglomeration was observed.  Figure 6 displays the effect of agitation speed on the removal of Cr(VI) ions by raw and metal oxides impregnated CNTs. The agitation speed was varied from 50 to 200 rpm, while the solution pH, initial concentration, adsorbent dosage, and contact time were kept constant at 6, 1 mg/L, 200 mg, and 2 hours, respectively. The removal of Cr(VI) ions was observed to increase with increase in agitation speed for all considered adsorbents. This is due to the fact that agitation facilitates effective diffusion of ions towards the adsorbent surface [73]. At 200 rpm speed, CNTs with 10% loading of iron oxide were able to remove 99% Cr(VI) ions.

### 3.6. Effect of Initial Concentration

**Figure 7:**
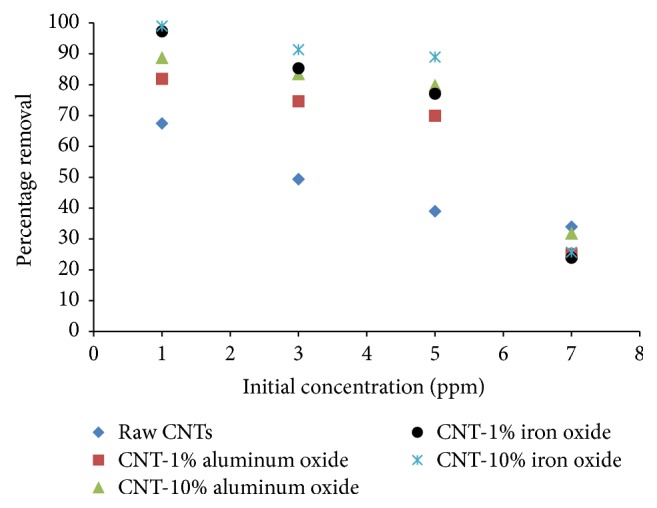
Effect of initial concentration on percentage removal of Cr(VI) (adsorbent dosage = 200 mg, contact time= 2 hours, agitation speed = 200 rpm, and pH = 6).

The removal of Cr(VI) ions was also dependent on the initial concentration of Cr(VI) as shown in Figure 7. The initial concentration was varied from 1 to 7 ppm, while the solution pH, agitation speed, adsorbent dosage, and contact time were kept constant at 6, 200 rpm, 200 mg, and 2 hours, respectively. The maximum removal was achieved at low dosage concentration and the removal was observed to decrease with increase in concentration for all adsorbents. This might be due to the fact that, at high concentration, the adsorption sites are saturated due to availability of surplus Cr(VI) ions. At 1 ppm dosage, a maximum removal 99% of Cr(VI) ions was achieved with CNTs with 10% loading of iron oxide.

### 3.7. Freundlich and Langmuir Isotherm Models

Adsorption equilibrium data was fitted by Langmuir and Freundlich models. Langmuir model best describes the monolayer adsorption while Freundlich model provides information about heterogeneous adsorption on adsorbent surface [81].

Representative equations of the isotherm models are presented below.

Langmuir isotherm model:

(4) $$\begin{array}{c} q_{e}=\frac{q_{m}K_{L}C_{e}}{1+K_{L}C_{e}}; \end{array}$$

Freundlich isotherm model:

(5) $$\begin{array}{c} q_{e}=K_{F}{C_{e}}^{1/n}, \end{array}$$

where *C*_*e*_ and *q*_*e*_ are the concentrations of contaminants in water and in adsorbent at the adsorption equilibrium, respectively. *q*_*m*_ is the maximum adsorption capacity; *K*_*L*_ is the adsorption equilibrium constant of Langmuir model; *K*_*F*_ and *n* are Freundlich constants related to the adsorption capacity and surface heterogeneity of the adsorbents, respectively.

**Figure 8:**
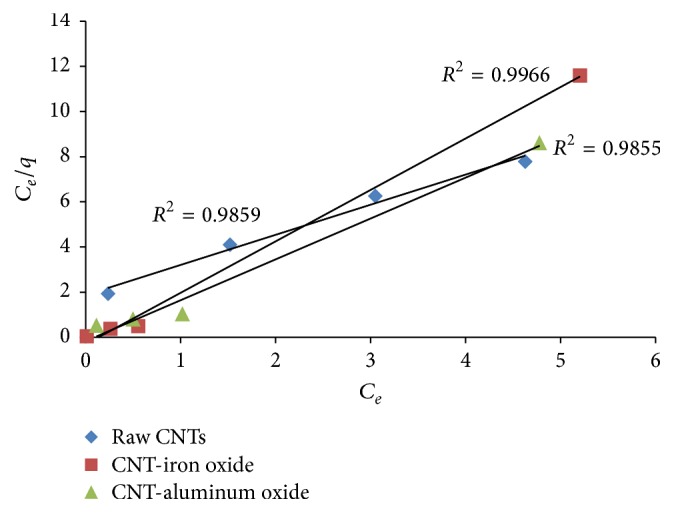
Langmuir adsorption model for Cr(VI).

**Figure 9:**
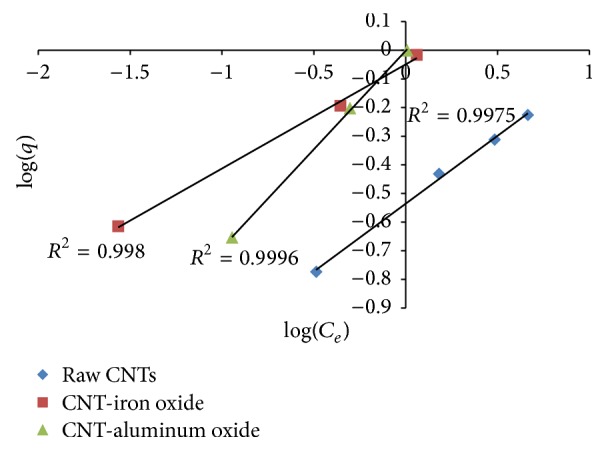
Freundlich adsorption model for Cr(VI).

Figures 8 and 9 show Langmuir and Freundlich adsorption isotherm models for Cr(VI), respectively, while adsorption parameters and regression data of the models are presented in Table 1. It can be seen that both Langmuir and Freundlich isotherm models show a good fit for both raw and metal oxide impregnated CNTs. However, the value of regression coefficient (*R*^2^) value for Freundlich isotherm model is slightly higher than Langmuir isotherm model.

### 3.8. Kinetics Modeling

Adsorption kinetic is one of the most important factors that govern the solute uptake rate and represents the adsorption efficiency of the adsorbent. The pseudo-second-order model was used to model the kinetics of adsorption.

Representative equation of pseudo-second-order model is provided below:

(6) $$\begin{array}{c} \frac{t}{q_{t}}=\frac{1}{k_{2}q_{e}^{2}}+\frac{t}{q_{e}}. \end{array}$$

**Figure 10:**
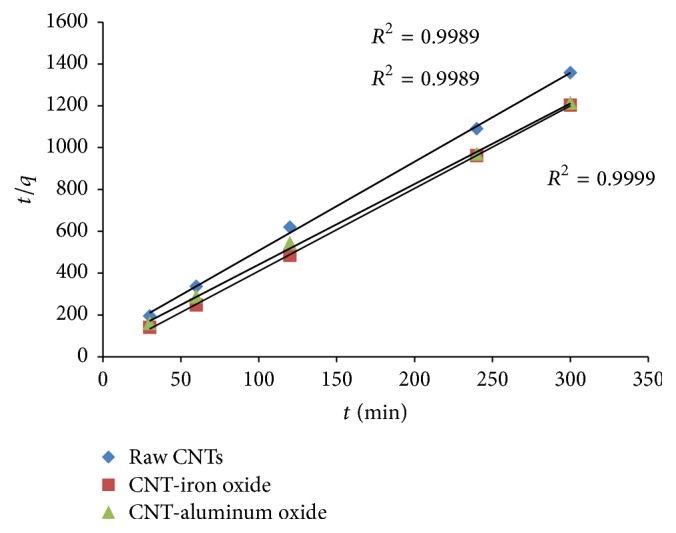
Pseudo-second-order kinetics for the adsorption of Cr(VI).

Figure 10 represents the fitting of experimental data with the pseudo-second-order model.  Table 2 provides the results of the kinetics model fittings for the adsorption of Cr(VI) using raw and metal oxide impregnated CNTs.

It can be seen from Figure 10 and Table 2 that the correlation coefficient (*R*^2^) of pseudo-second-order kinetic equation is sufficiently high for all the adsorbents. Therefore, the process of Cr(VI) removal using raw and metal oxide impregnated CNTs can be well described by the pseudo-second-order model.

## 4. Conclusion

Raw, iron oxide, and aluminum oxide impregnated carbon nanotubes (CNTs) were found to be effective adsorbents for the removal of Cr(VI) ions from aqueous solution. The removal of Cr(VI) ions was strongly dependent on pH, contact time, adsorbent dosage, and initial concentration of the Cr(VI) ions. Solution pH was found to be a critical parameter affecting the adsorption of Cr(VI) ions, in comparison with the other parameters. The removal of Cr(VI) ions was observed to decrease with increase in pH of the solution. It was observed that both CNTs impregnated with 10% of iron and aluminum oxides were able to remove almost 100% of Cr(VI) ions at solution pH 6, Cr(VI) initial concentration of 1 mg/L, adsorbent dosage of 200 mg, agitation speed of 200 rpm, and contact time of 4 hours. The prepared materials were found to exhibit high removal efficiency at pH 6 suggesting their great potential in real water treatment applications.

## Figures and Tables

**Table 1:** Parameters of Langmuir and Freundlich isotherm models for chromium.

Adsorbent	Freundlich			Langmuir	
	*n*	*K*_*F*_ (L/mg)	*R* ^2^	*K*_*L*_ (L/mg)	*R* ^2^
CNT-iron oxide	7.922564	0.628705	0.9980	−7.47535	0.9966
CNT-aluminum oxide	3.907029	0.571687	0.9996	−10.9559	0.9855
Raw CNTs	2.110755	0.291322	0.9975	0.756502	0.9859

**Table 2:** Parameters of pseudo-second-order kinetic model for chromium.

Adsorbent	*q*_*e*_ (mg/g)	*k*_2_ (mg g^−1^ min^−1^)	*R* ^2^
CNT-iron oxide	0.253062	0.534162	0.9999
CNT-aluminum oxide	0.259575	0.133789	0.9989
Raw CNTs	0.235297	0.109363	0.9989
